# Transoral robotic salivary surgery for hilar\parenchymal submandibular stones

**DOI:** 10.3389/fsurg.2024.1471207

**Published:** 2024-11-08

**Authors:** Pasquale Capaccio, Matteo Lazzeroni, Sara Torretta, Lorenzo Salvatore Solimeno, Valentina Cristofaro, Michele Proh, Giovanni Cammaroto, Giuseppe Meccariello, Claudio Vicini, Lorenzo Pignataro

**Affiliations:** ^1^ Department of Otolaryngology, Asst Fatebenefratelli Sacco, Milan, Italy; ^2^ Department of Biomedical, Surgical and Dental Sciences, University of Milan, Milan, Italy; ^3^ Department of Otolaryngology and Head and Neck Surgery, Fondazione IRCCS Ca’ Granda Ospedale Maggiore Policlinico, Milan, Italy; ^4^ Department of Clinical Sciences and Community Health, University of Milan, Milan, Italy; ^5^ Azienda USL Della Romagna, Morgagni Pierantoni Hospital, Forlì, Italy

**Keywords:** robotic surgery, submandibular, stones, sialolithiasis, sialendoscopy

## Abstract

**Objectives:**

A prospective interventional study was designed to describe our series of patients with submandibular stones undergoing sialendoscopy-assisted TORSS (trans-oral robotic salivary surgery) by means of Si or Xi Da Vinci robotic system between January 2019 and June 2023, in order to assess safety and effectiveness of the procedure.

**Methods:**

54 adult patients with submandibular stones undergoing sialendoscopy-assisted TORSS between January 2019-June 2023.

**Results:**

The global success rate was 81.5%, with better surgical outcomes in patients with palpable hilar/hilo-parenchymal stones compared to non-palpable pure parenchymal ones (92.7 vs. 46.2%). In addition, the mean stone size in cases failing TORSS was smaller than that documented in successfully treated patients (7.8 ± 1.8 vs. 9.8 ± 2.4 mm). No major untoward effects were observed (transitory lingual nerve dysfunction in 3 patients undergoing Xi Da Vinci surgery). A positive outcome in terms of post-operative surgical pain, patient's satisfaction and recovery time was observed.

**Conclusions:**

Intrinsic stone features (such as size, location/palpability) seems to be predictor for surgical success; an accurate pre-operative planning is mandatory to better select which patient can benefit most from TORSS procedure.

## Introduction

Until recently, treatment of obstructive salivary gland disease involved a transoral surgical approach in the case of submandibular stones located in the mid-distal portion of Wharton's duct or the transcervical surgical gland removal in case of deeper stones or unsuccessful conservative treatment such as interventional sialendoscopy (1–6).

Today, the rate of gland removal has been significantly reduced <5% and the development of a variety of minimally invasive techniques has led to a fundamental change in therapeutic perspectives (4, 7): sialendoscopy, alone or in combined endoscopic and incisional approach for larger stones, plays an essential role in the management of salivary gland benign pathology (4, 8). The conservative transoral approach, preserving the Wharton’s duct in its entirety, is used and it is now considered the gold-standard treatment for the treatment of hilo-parenchymal salivary stones (9).

The recent diffusion of transoral robotic surgery has favoured its application not only in the oropharyngeal and hypopharyngeal-laryngeal fields, the anatomical sites of major use of the method, but also in the oral floor for the removal of neoplasms and other pathologies of the salivary glands (10). By this approach, the risk of the most common complications of traditionally performed transcervical surgery is lowered, maximizing the possibilities of gland preservation (11).

Up to date transoral robot-assisted management of submandibular gland stones and transoral robotic submandibular gland removal have been described in case reports and small, heterogeneous series of patients (12–16).

The aim of our study is to describe a different approach to hilo-parenchymal stones of the submandibular gland by means of transoral robotic surgery and to evaluate safety and effectiveness of sialendoscopy-assisted TORSS procedure performed by means of two different robotic system in the treatment of patients with submandibular stones, by presenting out 3-year experience.

## Materials and methods

### Patients

The study included 54 prospectively recruited adult patients with hilo-parenchymal, hilar, or parenchymal submandibular stones undergoing sialendoscopy-assisted TORSS at the ENT Unit of Fondazione IRCCS Cà Granda—Ospedale Maggiore Policlinico (Milan) or G.B. Morgagni—L. Pierantoni Hospital (Forlì) from January 2019 to June 2023.

All the procedures performed in Milan were conducted by the use of with Xi da Vinci (Intuitive Surgical, Sunnyvale, CA, USA) robotic model; interventions that had taken place in Forlì were performed by means of Si da Vinci (Intuitive Surgical, Sunnyvale, CA, USA) robotic model.

The study was approved by the appropriate local ethics committee CEIIAV (Comitato Etico IRST IRCCS AVR Meldola, approval n. 1335, prot. n. 2587\2015) and was performed in accordance with the principles stated in the Declaration of Helsinki. The patients gave informed consent to participate in the study. A surgical consent for TORSS (always including possible conversion to traditional transoral stone removal) was administered in all the patient; in addition, in patients with non-palpable pure parenchymal stones, a surgical consent for possible conversion to (tranoral\transcervical) submandibular sialadenectomy was administered, too.

The stone was defined as hilar, hilo-parenchymal, or non-palpable pure parenchymal as previously reported (17–19).

Exclusion criteria were: presence of iatrogenic stenosis of Wharton’s duct; multiple intraparenchymal submandibular stone; acute submandibular gland inflammation, previous surgery or radiotherapy in the involved area, buccal opening inferior to 3.5 cm, macroglossia and micrognathia.

## Interventions

### Pre-operative assessment

All the patients underwent salivary glands ultrasonography and Doppler ultrasonography assessment (Hitachi H21, 7.5 MHz, Hitachi High Technology Corporation Ltd., Tokyo, Japan) and cone-beam 3D-CT (CBCT) of the lower mandible (CBCT 3D CS 9300 Carestream, Rochester, New York, USA) to define the number of stones, localization (hilar, hilo-parenchymal, pure parenchymal) and their size.

### The docking phase

**Figure 1:**
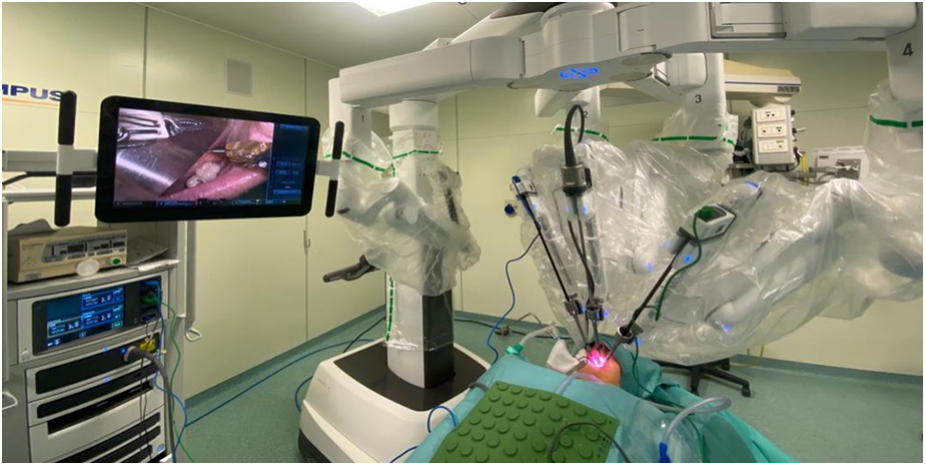
The Da vinci Xi robotic system docking phase—the camera is inserted into the middle arm, then the two instruments (spatula and Maryland forceps) in the side arms.

• *Da Vinci Xi docking:* The Xi robot is positioned to the right of the patient at 90° to the operating bed. After excluding the arm closest to the boom, the trocars are inserted into the remaining three active arms. First, the camera is inserted into the middle arm, then the two instruments (spatula and Maryland forceps) in the side arms. At this point, the camera position is set in the area of interest (oral floor) and the main surgeon places the instruments in the surgical field, while a square-shaped retractor pushes the tongue contralaterally to the operating submandibular gland and flattens the oral floor (Figure 1).
• *DA Vinci Si docking:* The Si robot is usually docked behind the head of the patient at an angle of 30° and to improve the visualization of the surgical field a 30° downward facing endoscope was placed in the scope-holder. Then, the usual robotic tools are put inside the respective trocars and a square-shaped tongue retractor, covered by a rough gauze, is settled into the patient’s mouth to push the tongue contralaterally and flatten the oral floor. At this point, surgery can start.

#### The surgical technique

After positioning the lateral mouth opener and once the docking phase is over, the first operator sits at the robotic console; using the monopolar spatula, he makes an incision in the oral floor of a few centimetres in the retropapillary-retromolar direction, while the assistant—using a tongue depressor—lateralises the lingual body contralaterally trying to flatten the involved oral floor as much as possible. The Warthon's duct and lingual nerve are identified and dissected on both the lingual and mandibular sites. The magnification and high image quality provided by the robot allow the robotic surgeon to perform these manoeuvres in a very short time, preserving as much as possible the function of these two anatomical structures.

**Figure 2:**
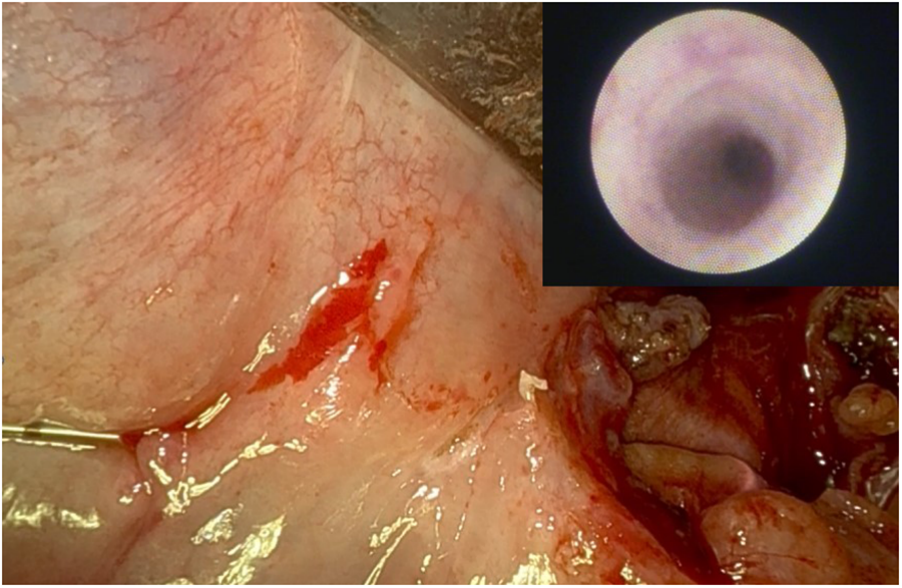
Incision of the submandibular gland's parenchyma to favour the release of the stone by means of Da vinci Si robotic model.

**Figure 3:**
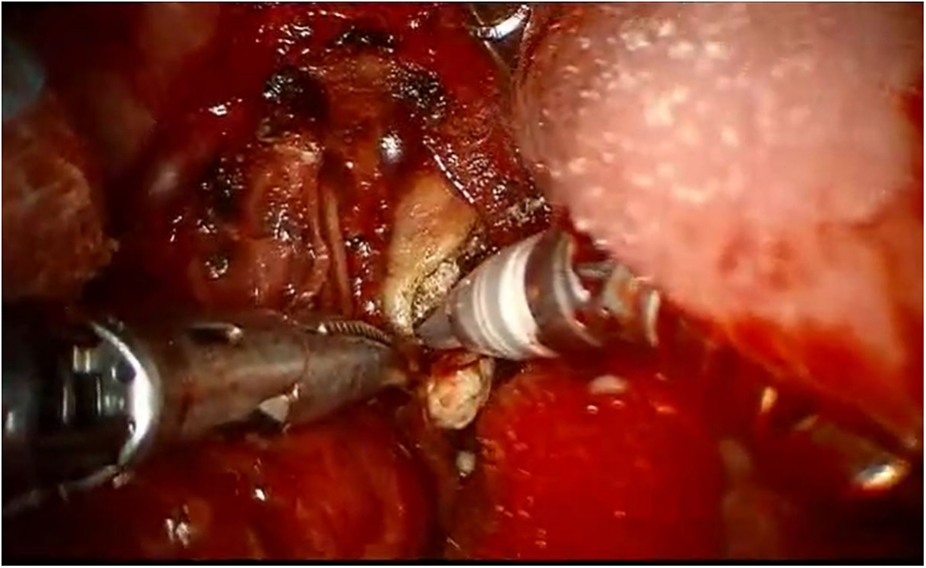
Removal of a right hiloparenchymal submandibular stone by means of Da vinci Xi robotic system.

Subsequently, depending on the position of the stone (hilar, hilo-parenchymal or parenchymal), the proximal portion of the duct is dissected down to the affected area. In the meantime, the third operator performs the push-up manoeuvre of the submandibular gland so that it can be superficialized in the oral floor. The first operator, once the position of the stone has been identified, incises the hilum or the parenchymal tissue covering it and removes it with the help of the spatula (Figures 2, 3).

At the end of the robotic procedure, the arms are undressed and the surgical breech is sutured with 3/0 or 4/0 resorbable thread after placement of a sterile resorbable Tabotamp® hemostat.

**Figure 4:**
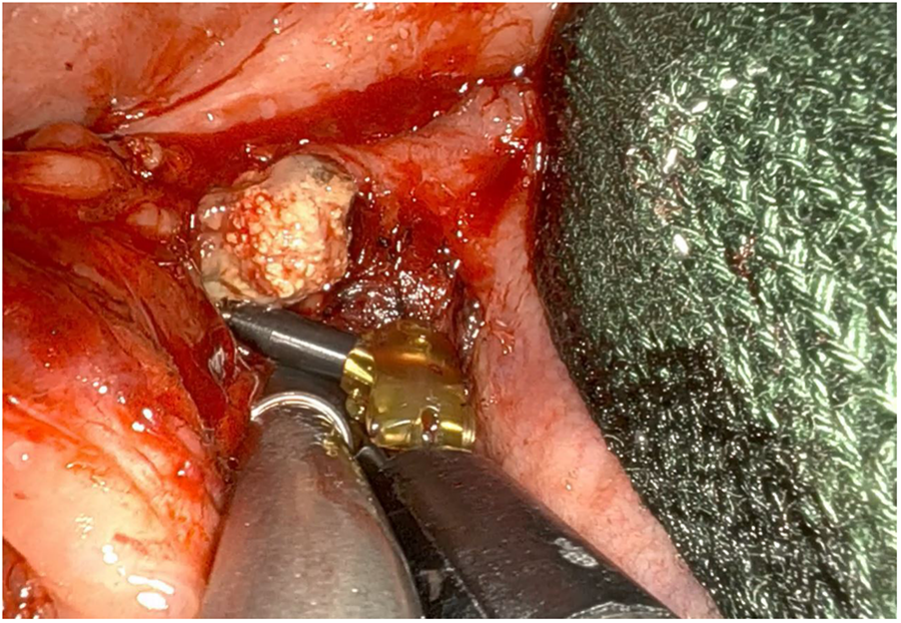
Post-operative sialendoscopy up to the hylum, transillumination and endoscopic view of the sialoendoscopic unit into wharton's duct.

After the procedure a post-operative sialendoscopy (0.8 mm Erlangen sialendoscopes, Karl Storz Co., GmbH, Tuttlingen, Germany) up to the hilum was done by our residents (ML, LS, VC) to check for residual microliths (Figure 4).

#### Post-operative assessment

All patients were treated with postoperative antibiotic therapy based on amoxicillin and clavulanic acid (1 gram 3 times a day for 7 days) and pain relief (paracetamol 1 gram as needed). Resumption of oral feeding occurs on the first day with the intake of liquid diet; solid foods are introduced starting on the second postoperative day, based on clinical condition.

All the patients underwent a Quality-of-Life questionnaire based on visual analogue pain scale (VAS pain) as previously described (18) the day after surgery, 7 days and 30 days after the surgical procedure.

Post-operative clinical and US assessment was performed 1 week, 1 month, 3 months, and then 1 year after the surgical procedure, in order to exclude any recurrence.

### Statistical analysis

Demographic and clinical features (including age, gender, location of the stone, docking time, and surgical time, VAS pain) were transcribed on an Excel electronic database (Excel 2016 v16.0, Microsoft Corporation, Redmond, WA), and data were extracted using the same program. The results are given as absolute numbers and percentages, or arithmetical mean values ± standard deviation (SD).

The procedure was considered successful in the case of complete stone removal during sialendoscopy-assisted TORSS. The location of the stone within the submandibular gland (i.e.,: hilar, hilo-parenchymal, parenchymal), and its characteristics (i.e.,: palpable vs. non-palpable) were analysed as possible confounders. Safety of the procedure was estimated by considering the rate of untoward effect.

## Results

The final analysis was based on the findings related to 54 patients including 33 (61.1%) males with a mean age of 53.3 ± 12.6 years (Table 1). All patients showed unilateral submandibular stones.

**Table 1:** Clinical and demographic features of the study population.

Characteristics		
Mean age (SD)		53.3 (12.6)
N°. of males (%)		33 (61.1)
Location (%)		
	Hylar	4 (7.4)
	Hylo-parenchymal	37 (68.5)
	Parenchymal	13 (24.1)
Side (%)		
	Right	23 (42.6)
	Left	31 (57.4)
Mean stone diameter (SD)		10.0 (3.7)
Successful (%)		
	Yes	44 (81.5)
	No^b^	10 (18.5)
Mean docking time (SD)		10.1 (2.6)
Mean surgical time (SD)		75.6 (42.8)
Mean VAS pain (SD)		
	Day 1	6.0 (3.6)
	Day 7	3.4 (2.2)
	Day 30	0.5 (1.3)
No°. with temporary lingual nerve impairment (%)		3 (5.5)
No. with other complications (%)^a^		3 (5.5)

^a^ 2 patients with infectious sialadenitis, 1 patient with oral floor oedema.

^b^ 8 patients undergoing contextual transoral stone removal; 1 patient undergoing trans-oral submandibular sialadenectomy; 1 patient undergoing transcervical submandibular sialdenectomy.

In most (68.5%) of cases, the stone was hilo-parenchymally located, while 24.1% of the stones were non-palpable pure parenchymal, and the remaining 7.4% were placed at the hilum (Table 1).

Sialendoscopy-assisted TORSS was successful in 44 out of 54 (81.5%) cases: in the remaining 10 cases, complete stone removal was achieved by contextual traditional transoral surgery (8 cases); in the remaining two cases also transoral surgery was ineffective, and then a trans-cervical submandibular sialadenectomy (1 patient) and a trans-oral submandibular sialadenectomy (1 patient) were performed. In one patient, sialendoscopy check at the end of TORSS achieved the visualization of a residual peri-hilar microlith which was successfully retrieved by means of an operative basket.

Considering the characteristics of the stone, sialendoscopy-assisted TORSS was unsuccessful in 7 out of 13 (53.8%) non-palpable pure parenchymal stones compared to 3 out of 41 (7.3%) palpable (hilar/hilo-parenchymal) stones. Among the seven failures occurring into the group of patients with non-palpable pure parenchymal stones, 5 out of 7 (71.4%) were conservatively surgically managed by means of traditional transoral stone removal; in the remaining two cases (2 out of 7; 28.6%) it was necessary to convert to transoral (1 patient) or transcervical (1 patient) submandibular sialadenectomy. All the three failures occurring into the group of patients with palpable hilar/hilo-parenchymal stones (due to partial stone removal during TORSS) were effectively managed by means of traditional transoral stone removal.

With regards the stone size, it is found to be smaller in cases unsuccessfully managed by means of sialendoscopy-assisted TORSS compared to the ones completely removed by means of primary robotic surgery (7.8 ± 1.8 mm vs. 9.8 ± 2.4 mm).

No major complications occurred, as only transient lingual nerve impairment (3 out of 54 patients, 5.5%), infectious sialadenitis (2 out of 54 patients, 3.7%), and mild oral floor oedema (1 out of 54 patients, 1.8%) were reported. Interestingly, all the patients with post-operative transitory lingual nerve impairment had undergone TORSS by means of DaVinci Xi system. However, in all but one case (experiencing mild lingual hypoesthesia till one year after surgery) a complete recovery occurred a few weeks after surgery.

A favourable outcome was also attested in terms of patient's satisfaction and recovery time, as 37 out of 54 (68.6%) patients were discharged the first day after surgery, 42 patients did not report pain on the VAS pain after a period of 30 days from surgery. All the interviewed patients would recommend this surgical procedure to other patients suffering from the same condition.

During the follow-up period (mean 13.4 ± 9.7 months), three out of 54 (5.5%) patients experienced recurrent obstructive sialadenitis without ultrasonographic evidence of residual stones, all but one adequately managed by means of medical treatment alone. In the remaining case, the patient underwent traditional submandibular sialadenectomy. In one patient (1.8%) a residual hilo-parenchymal stone was identified at ultrasonographic follow-up examination and removed by means of traditional transoral surgery.

## Discussion

Recently, transoral robotic surgery (TORS) has become a valuable approach in head and neck cancer surgery, and it has been progressively adopted also for anterior oral floor diseases (11). Sialendoscopy check up to the hilum performed at the end of the procedure is advisable to enhance the therapeutic effectiveness, as it achieves the identification and removal of possible residual microliths.

Initial TORSS experiences with stones have been performed with the Da Vinci robotic system (10). Its limitations are the rigid and relatively bulky robotic arms, a limited number of cutting devices available, and high costs.

Our results confirm our previous experience (14, 20, 21) documenting the effectiveness of sialendoscopy-assisted TORRS for submandibular stone removal, given a global success rate of 81.5%. In 9 out of 10 cases, the surgeon turned to a traditional one because the stones were deeply located inside the submandibular gland and the patients had reduced buccal opening not allowing optimal robotic arm movements (8 converted to traditional transoral stone removal, and one to transoral surgical sialadenectomy). In the remaining case, a transoral robotic-assisted submandibular sialadenectomy was effectively performed. Traditional transoral stone removal as a rescue treatment after TORSS failure may be needed in case of impossibility to intraoperatively detect the stone for non-palpable pure parenchymal stone, or in case of fragmentary partial stone removal during TORSS in patients with palpable hilar/hilo-parenchymal stones. With regards to this, the routinely use of CB-TC in the pre-operative diagnostic planning achieves a three-dimensional stone visualization, with clear definition of its shape and size. This piece of information is very important to be compared to the intra-operative findings, in order to be sure that the stone is completely retrieved during TORSS. Under these conditions, it is mandatory an accurate pre-operative counselling (with double surgical consent administration), in order to make the patient aware of the possibility of surgical conversion to submandibular sialadenectomy, in case of impossibility to intra-operatively detect a non-palpable pure parenchymal stone.

The global success rate here detected is slightly reduced compared to that reported by literature, and ranging between 91.9 to 100% of cases (14, 20, 21); in particular, Wen et al. (20) reported a 91.9% success rate in 24 patients, all with palpable >5 mm stones undergoing TORSS-assisted combined procedure. This difference probably lines within different patient selection criteria: as fact, in our case-series, after data stratification, we found a better surgical outcome in case of palpable hilar/hilo-parenchymal stones than in case of non-palpable pure parenchymal ones, with a success rate in the former sub-group of patients of 92.7% that sharply drops to 46.2% in the latter one. Under this condition, therapeutic effectiveness of sialendoscopy-assisted TORSS for palpable hilar/hilo-parenchymal stone completely lines within data reported by literature.

Based on this, and considering the corresponding figures for traditional trans-oral stone removal (98.5%) (18), an accurate pre-surgical staging considering the above mentioned features should be desirable in order to identify which patients could be more effectively managed by means of sialendoscopy-assisted TORSS (also considering non-negligible direct and indirect TORSS costs). The better three-dimensional magnification of the surgical field during TORSS can achieve some advantage over traditional surgery in case of deep locations. In addition, we believe that this therapeutic gap could be reduced by the routinely use of CB-TC a mean to objectively assess the precise stone location.

The difference in success rate by considering the site and characteristics of the stones (i.e.,: pure parenchymal vs. hilar/hilo-parenchymal; palpable vs. non-palpable; stone size) probably lines within the fact that during TORSS the advantages deriving from the tactile manoeuvre typical guiding the surgical dissection through stone identification during traditional transoral surgery are abolished. This hypothesis is supported also by the intra-operative detection of stones with smaller diameter (presumably more difficult to be intra-operatively located) in cases unsuccessfully managed by means of TORSS compared to the ones completely removed by means of TORRS. In our cohort, the mean stone size of 10.0 ± 3.7 mm was lower than that reported in other case-series: 12.4 and 12.3 mmm respectively in Wen et al. (20) and Razavi et al. ’s (21) studies.

Our results also confirmed the safety of sialendoscopy-assisted TORSS procedure, as no major complications occurred. As a fact, the most frequently reported untoward effects were a transient mild lingual nerve impairment (5.5%), completely resolved within a few weeks in all but one patient (who experienced a complete recovery by one year later), and sialadenitis (3.7%). The fact that all the patients reporting transitory lingual nerve dysfunction had been operated by means of Da Vinci Xi System, make us argue that the larger width of the surgical spatula in the Xi System compared to Si System (i.e., 8 vs. 5 mm) can results in a more traumatic effect on the delicate nerve structures placed in the anfractuous and restricted oral floor. In addition, the procedure resulted to be adequately tolerated by the patients, with an overall low morbidity attested by the high rate (68.6%) of hospital discharge on the first day after surgery, and the reported VAS pain on the first (6.0 ± 3.6) and the seventh (3.4 ± 2.2) days after surgery.

Beside costs and peculiar stone characteristics (i.e.,: location and palpability), the duration of the procedure (docking and pure surgical time) should be counted among possible disadvantages of TORSS: in our case-series the mean docking and surgical times were respectively 10.1 ± 2.6 and 75.6 ± 42.8 min, with a large variability (range 35–160 min) in surgical times based on surgical outcomes (64.2 ± 23.5 vs. 85.0 ± 32.5 min respectively in case of TORSS successful and failure) (data not showed). Compared to other case-series, our mean operation time (75.6 ± 42.8 min) is quite lower than that reported by Wen et al. (20) (103 min on average), being however within the range (62–120 min) (11, 14, 20, 21) of values described by literature. This probably is related to the improving surgical skills acquired during a period of three years from our two senior surgeons (i.e.,: PC and CV) working in two large University Hospital Centers used to perform major surgery, and taking advantage of robotic surgery skills acquired in other areas of the head and neck surgery.

The duration of the procedure (along with the probability of therapeutic success) considerably impacts on the cost-effectiveness of the approach, and it is primarily affected by three main factors, that are the experience of the surgeon, the location of the stone and specific anatomic insights. Regarding this last condition, an accurate pre-operative ENT assessment should be carried out to detect some possible red flags for TORSS: identification of hilo-parenchymal stones or proximally-located stones in a patient with unfavourable anatomy (i.e., reduced mouth opening) can result into a hard robotic procedure, usually not allowing the right help from the assistant surgeon in exposing the surgical field. Eventually, thanks to the 3D-HD view of the robotic camera and to the ability of the robotic arms to perform micro-movement with a much higher precision than the human hands, the transoral robotic surgical procedure to remove the salivary stones can be ruled out, whichever the utilized system is.

## Conclusions

Despite sialendoscopy-assisted TORSS seems a safe and effective approach for submandibular stone removal, better surgical outcomes occur in patients with large, palpable and hilar/hilo-parenchymal stones. Accurate pre-operative patients’ stratification is mandatory to enhance the surgical effectiveness of sialendoscopy-assisted TORSS procedure.

## Data Availability

The raw data supporting the conclusions of this article will be made available by the authors, without undue reservation.
